# Healthcare provider and patient perspectives on diagnostic imaging investigations

**DOI:** 10.4102/phcfm.v7i1.801

**Published:** 2015-05-20

**Authors:** Chandra R. Makanjee, Anne-Marie Bergh, Willem A. Hoffmann

**Affiliations:** 1 Faculty of Health Sciences, Department of Radiography, University of Pretoria, South Africa; 2 MRC Unit for Maternal and Infant Health Care Strategies, Faculty of Health Sciences, University of Pretoria, South Africa; 3 Department of Biomedical Sciences, Tshwane University of Technology, South Africa

## Abstract

**Background:** Much has been written about the patient-centred approach in doctor–patient consultations. Little is known about interactions and communication processes regarding healthcare providers’ and patients’ perspectives on expectations and experiences of diagnostic imaging investigations within the medical encounter. Patients journey through the health system from the point of referral to the imaging investigation itself and then to the post-imaging consultation.

**Aim and setting:** To explore healthcare provider and patient perspectives on interaction and communication processes during diagnostic imaging investigations as part of their clinical journey through a healthcare complex.

**Methods:** A qualitative study was conducted, with two phases of data collection. Twenty-four patients were conveniently selected at a public district hospital complex and were followed throughout their journey in the hospital system, from admission to discharge. The second phase entailed focus group interviews conducted with providers in the district hospital and adjacent academic hospital (medical officers and family physicians, nurses, radiographers, radiology consultants and registrars).

**Results:** Two main themes guided our analysis: (1) provider perspectives; and (2) patient dispositions and reactions. Golden threads that cut across these themes are interactions and communication processes in the context of expectations, experiences of the imaging investigations and the outcomes thereof.

**Conclusion:** Insights from this study provide a better understanding of the complexity of the processes and interactions between providers and patients during the imaging investigations conducted as part of their clinical pathway. The interactions and communication processes are provider–patient centred when a referral for a diagnostic imaging investigation is included.

## Abstrait

**Perspectives des prestataires de soins et des patients sur les examens d’imagerie diagnostique dans un complexe hospitalier sud-africain: une étude qualitative.**

**Contexte:** Beaucoup d’encre a coulé sur l’approche centrée sur le patient dans les consultations du docteur avec son patient. On sait très peu sur les interactions et les processus de communication en ce qui concerne les perspectives des prestataires de soins et des patients sur les attentes et expériences des examens d’imagerie diagnostique pendant la rencontre médicale. Les patients parcourent le système de santé du point de référence à l’examen d’imagerie même, puis à la consultation post-imagerie.

**Objectif et lieu:** Examiner les perspectives des prestataires de soins et des patients sur l’interaction et les processus de communication pendant les examens d’imagerie diagnostique dans le cadre de leur parcours clinique dans un complexe sanitaire.

**Méthodes:** Une étude qualitative a été menée avec une collecte de données en deux phases. On a sélectionné aisément vingt-quatre patients dans un complexe hospitalier public de district, puis on les a suivi tout au long de leur parcours dans le système hospitalier, depuis l’admission jusqu’à la sortie de l’hôpital. La seconde phase consistait en entrevues avec un groupe cible menées avec les prestataires de soins dans l’hôpital de district et l’hôpital universitaire voisin (agents médicaux et les médecins de famille, infirmières, radiographe, les consultants en radiologie et les registraires).

**Résultats:** Deux thèmes principaux ont guidé notre analyse: (1) perspectives du prestataire; et (2) dispositions et réactions du patient. Les fils conducteurs qui recoupent ces thèmes sont les interactions et les processus de communication dans le contexte des attentes, des expériences des examens d’imagerie et le résultat de ceci.

**Conclusion:** Les éclairages apportés par cette étude offrent une meilleure compréhension de la complexité des processus et interactions entre les prestataires et les patients au cours des examens d’imagerie effectués dans le cadre de leur parcours clinique. Les interactions et processus de communication sont centrés autour de la relation du prestataire avec le patient quand un examen d’imagerie diagnostique est inclus.

## Introduction

Referral for a diagnostic imaging investigation is not an isolated event but is rather an integral part of a complex medical encounter that often involves interaction with multiple healthcare providers and technologies. A patient-centred approach to justifying a referral for and conducting a diagnostic imaging investigation entails knowing the patient as a person, engaging with and listening to the patient as an active participant and providing quality professional services.^1^ Diagnostic reasoning not only comprises an analytic process, but also involves an affective component.^2^

The demand for greater patient-initiated access to medical^3^ and imaging^4,5^ services has grown, especially as a result of the consumerist movement.^6,7^ Failure to meet patient needs or requests impacts on visit satisfaction and patients’ health-related anxiety increases when the desired diagnostic intervention is not received.^8^ However, patient centredness does not imply giving a patient what he or she wants;^9^ uncritical compliance with such requests is both unprofessional and unethical.^8^ Therefore, effective communication is essential in patient-centred medical practice in order to be able to give the necessary priority to patient safety.^10^

Referral for a test could increase patient concern and fears that the symptoms indicate a serious illness.^11,12^ Patients referred for a diagnostic imaging investigation are often in a vulnerable state^13^ and their anxiety and discomfort may contribute to poor patient satisfaction.^14^

There are numerous research reports on patient participation in clinical decision making. However, not much has been written on interactions and communication processes between healthcare providers and patients and amongst different providers regarding diagnostic imaging investigations, nor have these processes been positioned within the broader context of the medical journey from admission to referral and discharge.

### Aims and objectives

The aims of this study were to explore how patients expressed and positioned themselves and how they changed their perceptions in the period between pre- and post-diagnostic imaging; and how healthcare providers perceived patient expectations and their ability to participate in a medical encounter.

## Research methods and design

This study represents one of the first attempts to explore multi-perspective decision making and interactions in diagnostic imaging investigations. A qualitative research design with two consecutive phases was used. The first phase entailed shadowing patients from admission to discharge, whereas the second phase consisted of focus group interviews with healthcare providers.

### Study design

The study was conceptualised around the metaphor of a patient’s journey through the hospital system (admission through to discharge), in the context of accessibility of diagnostic imaging investigations. The various healthcare providers with whom the patient interacts along the journey form part of the organisational culture of the healthcare institution. Upon arriving at the hospital, the patient connects with nurses and doctors. During the medical consultation, the doctor makes a decision regarding a diagnostic investigation referral. At the imaging department, the patient then interacts with radiographers and/or radiologists before returning to the referring medical practitioner or specialist.

### Setting

This study was conducted at an urban South African district hospital that is part of an academic complex including a primary healthcare (PHC) clinic, a provincial tertiary hospital and a central hospital. The complex provided the whole spectrum of imaging services with a system of upward and downward referral according to choice of imaging modalities provided at the different levels of care. Patient journeys started at a PHC clinic or at the casualty or outpatient (OPD) departments of the district hospital.

### Sampling

Twenty-four patients were recruited for phase one through convenience sampling. The inclusion criteria were the ability to give consent, as well as a willingness to communicate with the researchers in English or through an interpreter and to spend an extra half hour after completion of the last medical consultation. Under-18 and critically-ill patients were excluded. Patient participants came mostly from poor socioeconomic communities. Table 1 provides an overview of these participants.

**TABLE 1:** Overview of patient participants.

Characteristic	Breakdown	*n*
Hospital entry route	Direct	15
	Referral	9
Origin of referral	Self	15
	Clinic	3
	Casualties	1
	Private	4
	Follow-up	1
Admission department	Casualties	14
	Outpatients	10
Referral for diagnostic imaging	Yes*	18
	No	6
Gender	Male	7
	Female	17
Age (years)	Mean	40
	Median	45
	Range	18–83

*, General x-rays only (*n* = 12); General x-ray and CT (*n* = 2); Stereotactic breast biopsy and mammography (*n* = 1); Diagnostic ultrasound (*n* = 2); Gynaecological ultrasound conducted by medical practitioner (*n* = 1).

Healthcare providers were recruited for both phases of the study. In the first phase, they were interviewed individually following the medical consultations. The providers included: nurses; radiographers; medical students; interns; community service doctors; medical officers; family physicians; radiology consultants and registrars; and specialist consultants and registrars.

For the second phase, a purposive sample of healthcare providers was recruited to participate in focus group interviews. Some providers participated in both phases of the study, whereas others only participated in either phase one or phase two.

### Data collection methods

Table 2 and Table 3 provide details of the data collection process and methods used in the two study phases. The study design for phase one included researcher observations of the patient-provider interactions at all points of medical and imaging care services. Most inter­actions were accompanied by an audio-recording and the attending researcher made field notes.

**TABLE 2a:** Data collection process and methods - Phase I: Data sources for each patient.

Steps	Patients (*n* = 24)	The journey	Providers (*n* = 62)
Step 1: Pre-consultation	Entry interviews ( = 24) (audio-recordings )	-	-
Step 2: Medical encounter	-	Doctor–patient consultations (*n* = 19) (observations, audio-recordings, medical files & field notes)	-
Step 3: Diagnostic imaging	-	Radiographer–patient interactions (*n* = 17) (observations, request forms and field notes)	-
Step 4: Post–imaging	-	Doctor–patient consultations (*n* = 17) (observations, audio-recordings, medical files & field notes)	-
Step 5: Discharge	Exit interviews (*n* = 22) (audio-recordings)	-	-
Step 6: Provider interviews	-	-	Medical practitioners (*n* = 20) Radiographers (*n* = 18) Radiologists and registrars (*n* = 17) Other specialties (*n* = 4) Nurses (*n* = 3) (audio–recordings)

**TABLE 2b:** Data collection process and methods - Phase 2: Provider focus groups (audio-recordings and field notes).

Profession	Focus groups (*n* = 12)	Participants (*n* = 53)
Medical practitioners and family physicians	3	13
Radiographers	3	15
Radiologists and radiology registrars	2	8
Nurses	4	17

We conducted individual, semi-structured interviews with patients at the entry and exit points, as well as individual interviews with healthcare providers involved with each of the patient participants. Most interviews were held in English, with interpretation for two patients. Entry interviews with patients were conducted before consultation with the medical practitioner. The interviews probed patients’ reasons for their visit, their expectations and their specific knowledge of diagnostic imaging. Exit interviews were conducted after they had received a treatment plan from the attending medical practitioner or specialist. These interviews focused on whether their expectations had been fulfilled regarding the care they had received during their hospital journey.

The individual interviews with healthcare providers probed the following aspects of the specific patient consultation and interaction: referral decision and justification; outcome(s) of the diagnostic imaging investigation(s); and approach to engaging with the patient. Although the ideal was to follow (shadow) all patients at all times, in two cases the medical providers changed their referral decisions, resulting in those patients being taken for x-rays without the presence of a researcher. Because of the unpredictability of discharge dates and admission times, follow-up exit interviews with two patients could not be conducted.

The phase two focus group interviews were conducted with 53 participants from different healthcare provider categories: medical practitioners and family physicians; nurses; radiographers; and radiologists and radiology registrars (Table 2a and Table 2b). Issues addressed in the focus groups included patient expectations and experiences of referrals, as well as provider experience of accessibility to diagnostic imaging services.

### Data analysis

**FIGURE 1:**
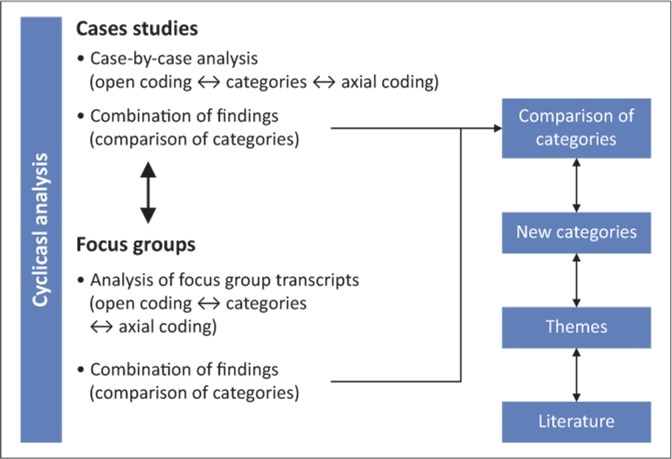
The data-analysis process.

All audio-recordings were transcribed. One researcher (C.R.M.) conducted the first round of data analysis manually. This was followed by several consensus discussions by the research team. Figure 1 illustrates the data analysis process. The phase one data analysis followed a ‘bottom up’ (p. 38)^15^ approach in which the data from each patient case (including observations and provider interviews) were analysed by coding for categories. Data were organised into four components. Data from the entry and exit interviews and observations were grouped together and analysed concurrently to inform the patient’s journey, whereas the providers’ individual interview data informed the provider perspective. Emergent codes for each patient case were compared, consolidated and expanded with the analysis of each subsequent patient case. At the next level of analysis, categories emerging from the patients’ journeys and those from the provider perspectives were compared and ultimately integrated in a comprehensive provisional structure of categories, subthemes and themes.

The phase one findings informed the content and structure of the focus group interviews. Data analysis of the transcripts of the focus groups followed the interviews. Member checking was performed by eliciting feedback from healthcare providers, but not from all patient participants because of the recurrent change in contact details or non-availability of participants after the exit interviews.

### Trustworthiness

The principles of confirmability, credibility, transferability and dependability were followed to ensure the trustworthiness of this study.^16^ The three researchers had different roles regarding their ‘insider’–‘outsider’ relationship with the research setting.^17^ Two researchers were outsiders (A.M.B., W.A.H.) and the third researcher (C.R.M.) was familiar with the setting and knew some of the radiographer participants but was not employed in the setting. This provided sufficient distance to allow the researchers to appreciate the patients’ journeys and the providers’ perspectives without jeopardising the confirmability of the study.

The credibility of the findings was enhanced by the active participation of all three researchers in data analysis and interpretation. At times they served as peer reviewers for each other with regard to the identification and integration of categories, subthemes and themes. Study findings were submitted for scrutiny to two independent healthcare experts familiar with the research setting, a radiologist and a family physician.

With regard to transferability, thick descriptions with verbatim accounts are used to enable readers to discern whether the findings are applicable to similar settings. To ensure dependability, the accounts of each patient case and the focus group data for the different healthcare provider categories were included in the data analysis process; deviant cases were also noted and taken into account.

Data collection triangulation in the form of individual interviews, observations of consultation sessions and focus groups ensured that the voices of patients and various healthcare provider categories were integrated in the study findings.

### Ethical considerations

This study formed part of a qualitative project on decision making and interactions in diagnostic imaging investigations. Ethics approval was granted by the Research Ethics Committee, Faculty of Health Sciences, University of Pretoria, South Africa (170/2008) and health managers gave written permission for access to the research sites. Signed informed consent was obtained from all research participants, which included strict assurances of voluntary participation and confidentiality. Five provider participants declined audio-recording of the patient-doctor consultation. All, except one of these, gave permission for the field researcher to be present and taking written notes during the consultation. Three of these providers chose not to be interviewed individually after the consultations.

## Results

The analysis centred around two main themes: (1) provider perspectives; and (2) patient dispositions and reactions. Table 3 provides a summary of subthemes and categories associated with each main theme. Direct quotations from the individual interviews and focus group interviews are provided, where appropriate, as supportive evidence for each subtheme. The following codes are used to refer to the different participant groups: [FG] = focus group; [II] = individual interview; [PI] = patient interview; [MP] = medical practitioner or family physician; [RADL] = radiologist or radiology registrar; [RAD] = radiographer; and [NP] = nurse. Where applicable, a number refers to the number of a particular patient participant.

**TABLE 3:** Summary of main themes, subthemes and categories.

Themes	Subthemes	Categories
Provider perspective	Perceptions of patient expectations	-
	Patterns of communication and consultations	Medical practitioners Nurses Radiographers Radiologists Communication of results
Patient dispositions and reactions	Expectations and reluctance to communicate	Unfamiliarity with x-ray investigations Wait-and-see
	Experiences of imaging investigation process	From anxiety/fear to relief The whole story

### Provider perspective

The two main emerging subthemes for providers are their perceptions of patient expectations and communication patterns with patients.

#### Providers’ perceptions of patient expectations

Medical provider participants often referred to the so-called ‘demand’ by patients created by the nature of some patients’ injuries, by observing other patients being referred for diagnostic imaging investigations, or by expectations raised by the referring centre. The following quote is illustrative:

‘There are two groups of patients … those who accept whatever the doctor says. They have come here for diagnosis. And those who made up their mind, they’re coming to confirm their suspicion. Those are the patients that will demand diagnostic investigations, even if they don’t need it. They demand and these are from the good socioeconomic status, the ones that are educated.’ (II: Neurosurgery registrar, Patient 7)

Another provider perception was that patients thought that pathology would be missed if they were not referred for diagnostic imaging investigations:

‘The problem is that the patient sometimes has funny ideas about what is wrong with them. … For instance, if the patient experiences a cough for a long time or for a week; it might just be a cold but [*the patient*] might think it’s cancer. … So for them not to be sent for x-rays … they don’t understand it, because we are going to miss their possible cancer. That’s what makes it difficult for patients.’ (FG:MP)

Medical practitioners were of the opinion that patients ‘think we can see everything on x-ray’ and expected to be healed by ‘this magical thing’ that is ‘going to change my life now forever’ (FG:MP), ‘like a treatment they’re going through’ (FG:RAD).

Healthcare providers’ perceptions that patients expected referrals for diagnostic imaging investigations resulted in them favouring the use of technology as a pacifier and a ‘quick fix’ to prevent ‘come backs’ (FG:MP):

‘But then they still go away thinking that lousy doctor did not send them for x-rays. Doctors are not doctors until they have done x-rays, given an injection and big fat packet of medicine and then they feel good. You think the patient is fine, but they think you actually look into their problems when you have an x-ray. It is actually psychological that you do something, even if it’s not 100% indicated. The patient feels you’re doing something, because the patient is worried. To prevent more visits.’ (FG:MP)

Other medical providers justified referrals for diagnostic imaging investigations out of moral obligation: ’Some of them [patients] claim from the road accident funds’ (FG:MP). Others were concerned about failing to make the correct diagnosis:

‘I think you can take a lot of x-rays … a little bit over-treating the patient. … I feel more comfortable doing … everything. I think there could be a possible fracture, more just purely on clinical [*grounds*], so if you’re doing one side, then you can miss something. Often when it comes to comparing, it is when you can see it.’ (II:MP, Patient 24)

#### Patterns of communication and consultations with patients

One of the communication challenges and concerns high­lighted by different providers was language comprehension and/or the less-than-ideal provision for language diversity between providers and patients:

‘Things get lost in translation. … I worry sometimes that maybe I ask a question, they don’t quite get the proper clinical picture. And maybe when I give the information, they don’t quite understand everything that I am saying.’ (II:MP, Patient 16)

##### Medical practitioners

The observation data collected during the initial provider-patient consultations indicated that medical practitioners tended to take the patient histories in the form of rapid question-and-answer sessions without offering patients sufficient time to elaborate on their responses. Similarly, decisions to refer patients for diagnostic imaging investigations mostly involved one-way, non-negotiated communication, without any significant discussion regarding the risks and benefits or what could be expected of the investigation. One practitioner admitted that ‘sometimes we forget to really talk to the patient’ (FG:MP).

Others assumed that patients were knowledgeable about x-rays:

‘That is where the doctors are lacking a lot. Because we don’t talk to the patients about it, the risk and it is dangerous and things like that.’ (FG:MP)

In most cases, patients were merely informed that they were going to be ‘sent for x-rays’ (PI:1), without seeking their approval or disapproval. Nurses and medical practitioners also alluded to system pressures and organisation of patient care encroaching on time available for patient interactions:

‘This is not a quiet clinic. I have seen 69 patients. I have got six clinics running … x-ray department closes at three. … Chemist close at quarter to four; if they’re not there they have to also return the next day. So it’s all time constraints that you’re working with.’ (II: NP, Patient 17)

Patients also observed the ‘time constraints’ medical practitioners referred to, saying that ‘the doctors do not give us the chance to talk to them; they are already on the move’ (PI:13).

Interprofessional blaming emerged during the study, specifically with regard to who should be responsible for providing patients with information regarding diagnostic imaging procedures. Radiographers and radiologists often blamed medical practitioners because ‘they don’t inform the patients what they’re going to do and who’s going to do it’ (FG:RAD).

##### Nurses

Nurses indicated that patients viewed them as a source of information at various points of contact, whereas the actual need for information varied from person to person:

‘Some [*patients*] don’t even know what x-ray [*is*]. They ask you, “What is x-ray? What are we going to do there? Is there any big machines? Am I going to feel the pain when they’re going to do this?” And then I try to explain to them … “You are going to stand there and they have got a big light and a big machine and they are going to take a photo of you. But this photo take of your inside, not your outside appearance.”’ (FG:NP)

‘Some of them don’t even ask. They just take the [*x-ray*] envelope, open it and start looking like this and they want you to start explaining to them.’ (FG:NP)

##### Radiographers

The observation data indicate that the imaging process resembled a production-chain setup. One radiographer admitted: ‘Sometimes I do not check … [*with*] the patient what’s going on’ (II:RAD, Patient 1). This is how one patient described her experience:

‘I just gave her [*radiographer*] the paper [*request form*] the doctor gave me. She took the paper. She took me next door [*to the x-ray room*]. She took the x-ray and after that she said I must sit on the bench. She came; she gave me the x-rays. “Go back to the nurses to give it there.”’ (PI:24)

There were, however, also patients who expressed appreciation for the radiographers’ interpersonal interactions, saying that ‘they were good; I observed how their attitude was and how they handled me’ (PI:5).

##### Radiologists

The interaction between radiologists and patients can mostly be described as a non-relationship:

‘We don’t have much contact with the patient on sort of day-to-day running of plain x-rays to see the patient.’ (FG:RADL)

This was also the case of our observations of patients referred for computerised tomography (CT) and ultrasound investigations. Patient 8, who shared her fear of losing her leg with the field researcher, had a very impersonal experience, with minimum interaction during her ultrasound investigation. The radiology registrar looked at the screen – not visible to her – without explaining anything and merely instructing her occasionally to elevate her leg.

##### Communication of results

In addition to the above lack of significant communication interactions, the researchers observed information download during post-imaging consultations where the patient was not afforded sufficient time to fully comprehend what had been said. In the majority of instances, healthcare providers did not involve the patients in the interpretation process, despite some patients showing interest in viewing their radiographs and wanting to be part of this. At this point, the issue of the ‘big secret’ (FG:RADL) arises. When Patient 21 tried to get a look at her x-rays the medical practitioner grabbed the x-rays and envelope, saying, ‘This is mine!’

Patients frequently expected information about their outcomes at the imaging site: ‘I would have liked to know whilst I was at the x-ray department’ (PI:10). On the other hand, providers also seemingly expected patients to initiate the information-seeking process:

‘That’s if the patient wants to see the investigation or the films then and they should have the right to ask. The referring clinician has to have a look at the films as well and I think it’s at that point that the patient should be shown the images.’ (FG:RADL)

### Patient dispositions and reactions

Patient expectations and reluctance to communicate, as well as patient experiences of the imaging investigation process are the two main subthemes that emerged from the analysis.

#### Patient expectations and reluctance to communicate

##### Unfamiliarity with x-ray investigations

Unfamiliarity could be one of the reasons for patients’ reluctance to communicate with the healthcare providers. Although most participants knew about x-rays, only a few had a good understanding of radiation and its effects on the human body. This is how a patient with acute right hypochondriac pain expressed his understanding:

‘No, you don’t have x-rays too often, maybe once every six years. You don’t have it a lot. X-rays are harmful because they’re radioactive. But cell phones are just as harmful.’ (IP:7)

The same patient was the only participant who explicitly verbalised a need for x-ray referral: ‘Well, just take x-rays of my [*abdomen*] … Show what’s up and what is going on’ (IP:7). Other patients were less vocal and expressed their expectations in more general terms such as ‘I want them to help me’ (IP:4). They often relied on or complied with the medical practitioner’s opinion – ‘If they [*the doctors*] say for an x-ray, I know I must go’ (IP:16).

##### Wait-and-see

During the doctor–patient consultation, patients tended to adopt an approach of ‘one looks at the situation before you can ask’ (IP:13). Most patients were reluctant to ask the medical practitioner for information as they did not feel comfortable with that or with divulging detailed information during history taking. In addition, hardly any opportunities were created for patients to ask for clarification or to express concerns – in a metaphorical sense, the patients’ voices were silenced.

‘The doctors don’t talk to you so much … but sometimes the doctor … was a harsh somebody … It might frighten me sometimes if I don’t know my story … I keep quiet about it. … Sometimes even before you give him the answer, he already answers it for you.’ (IP:17)

The inadequate sharing of information and referral decisions by medical practitioners with patients regarding the purpose of the referral and/or potential alternative diagnostic options was highlighted when those patients interacted with radiographers at the imaging department. One radiographer shared the following:

‘I had this experience where this patient came in for an [*barium*] enema. “Did they explain to you?” Then she said, no, she doesn’t want it; are there any alternatives? And then I said, “Maybe you could get a scope or a scan.” Then she wanted to go back to the doctor to get another second investigation. She refused the enema because she didn’t understand what she made that appointment [*for*].’ (FG:RAD)

#### Patient experiences of the imaging investigation process

One shared experience of patients journeying through the health system was the oscillation between fear or anxiety and feelings of relief. By the end of their journey, very few patients had adequate communication experiences.

##### From fear and anxiety to relief

Patient reactions during the exit interviews succinctly illustrated their fear or anxiety during the journey, as well as the ultimate relief experienced. On the one hand, they experienced fear of technology – the ‘cold environment’ (FG:NP) – at the diagnostic imaging department, whereas on the other hand, they experienced anxiety about the possible diagnosis.

Fear was clearly illustrated by a patient who had received inadequate information regarding what to expect from a stereotactic breast biopsy performed with imaging techniques. She was also frightened by the interactions that took place between the radiology registrars, radiographers and a nurse during the image-guided biopsy, but her journey had a good end:

‘I was feeling scared. … How are they going to do this thing? They say they are going to cut a piece of meat. It’s where my imagination started to make me scared because I thought they were going just to cut me like that. … They told me that they are going to inject me with a needle and they are going to cut me. … I was not sure [*how*] they were going to do that. Maybe they [*wanted to*] make me not to be scared. … I was not expecting it to be like this. It horrified me. … I didn’t even see the doctor [*radiology registrar*] that was doing the biopsy. … I thought those nurses are going to do it. … I know that in my family there is a cancer problem. … I was so scared until now … when they did do the results. … But today at the end I am very happy.’ (IP:13)

Inadequate preparation could lead to anxiety-provoking experiences for some patients and persons accompanying them when they had their first overwhelming encounter with ‘the big machine’:

‘The minute we got into the x-ray room, we saw these big machines, … [*my daughter*] started to cry. … I did not really prepare [*her*] for this psychologically, because she is three years old. … Everything is okay until we got to this cold room. … There was no talking. She [*the radiographer*] said, “Come in”; “Okay, hold her arm”; and that was it. … I felt neglected; you are not important. Just do this, that’s all. They don’t care. They just have to do their job. … You know, when you work with kids … that [*reassuring*] voice of yours. But there was nothing. “Okay, we’re finished. Okay, go. Bye.”’ (FG:NP, on her own experience)

At the conclusion of the diagnostic imaging examination, some patients expressed a sense of relief, even if it was based on a seemingly incorrect understanding of the aim and/or outcome of the actual examination:

‘Some patients really, they don’t understand what x-rays are all about. Because the patient comes in and you ask him if he can stand, he says, “No, I can’t stand.” And you go to the room and then the patient stands up, you do the PA [*postero-anterior projection*], you do the lateral. And so, “I feel much better now.” So he doesn’t even know what the x-ray is all about. He just thinks we’ve done this big thing on him, now he is feeling lighter than before or “I am feeling much better now.”’ (FG:RAD)

##### ‘The whole, whole story’

One may reasonably expect that patients at the end of their journeys would have a good idea of the underlying causes and/or explanations of their symptoms, the ultimate diagnosis and implications of their conditions, and the treatment plans that they should follow. Medical practitioners were of the opinion that it was ‘the duty of the attending physician to put everything together, tell the patient the whole story’ (FG:MP).

However, by not engaging in effective communication with patients and not confirming their comprehension level, patients can get lost in their journey and end up with inadequate information and understanding of their condition:

‘I have seen patients; they have been through the whole system. They come back and you ask them … “Have you got a fracture?” “No, I don’t know. They never told me.”’ (FG:MP)

Only two patients reported getting the ‘whole story’; one of them expressed it as follows:

‘It was really a benefit to me because even on the x-rays the doctor showed me. I like also the fact that the doctor showed me … in the book … how the whole thing started and it helped me as well. So I think the x-rays are a very good thing and what I like from that, I just know that I have sinus. Before when I go to the doctor, then they check. … They say my sinuses are blocked inside. So it’s the first time that I experience that they show me the x-rays; how even my eyes, I didn’t know that, the x-rays will take it out as well, as it’s part of the sinus, the infection of the eye. I know now the whole, whole story of the problem that I have. And then for that I know I will be more careful. For sometimes you know you mustn’t be in the place where people smoke, but still you are there. So now I know exactly that I must really strictly avoid those types of things … That is how it must go. The doctor must explain to you the cause of the sickness and show how the parts happen and what happens.’ (IP:12)

## Discussion

This study explored the expectations and experiences of patients regarding diagnostic imaging investigations from the point of consultation and referral up to the reporting of the results and outcomes of the imaging investigations within the medical encounter. The study also explored the various healthcare providers’ perceptions of patient expectations and patterns of communication and interaction.

The perceived patient expectations by medical providers are sometimes based in the providers’ own uncertainty, cautiousness and desire to confirm or exclude a diagnosis.^18^ In other cases, patients may demand referrals for diagnostic imaging investigations, as was found in a study by Baker *et al.*^19^ in which medical practitioners were under pressure to refer patients for spinal x-rays. Some medical practitioners in our study also reported similar demands, whereas some conceded to using technology as a pacifier – a ‘quick fix’ approach – to prevent come-backs. System pressures were sometimes experienced as a dominant factor in referrals; the emphasis was on throughput of relatively large numbers of patients per day. Similar to the study by Jayadevappa and Chattre,^20^ the current study found that patient satisfaction was not so much linked to their specific expectations but rather with the effective management of the condition and/or situation.

Communication gaps identified during the provider-patient interaction were limited two-way communication and low patient participation in the decision-making process at the time of the referral for a diagnostic imaging investigation, during the actual investigation and during the post-investigation consultation. A clear advantage of informing patients about the potential risks of imaging would enable them to make informed decisions at that moment and in the future regarding complex issues concerning their healthcare.^7^ Similar to Malone *et al.*’s findings,^3^ this study found hardly any evidence that information on radiation risks was mentioned or explained in the doctor–patient consultations or radiographer-patient interactions. Some of the medical practitioners in our study seemingly assumed that patients had an adequate knowledge of the radiation risks and benefits associated with diagnostic imaging investigations. Radiographers focused on possible risks during pregnancy and with the procedure task at hand. Reeves and Decker^21^ describe the image and not the patient as being the centre of the diagnostic radiography practice; the images distance the radiographer from the patients and their suffering. Murphy^22^ suggests that radiographers merely act as operators of equipment in a patient-unfriendly environment that leaves little room to actually listen and respond to patients’ information and support needs.

Patient participants’ general unfamiliarity with diagnostic imaging investigations and their reservations about initiating information-seeking dialogue with healthcare providers are reflected in their wait-and-see attitude. The reason for this ‘passive partner role’ (p. 578) is aptly described by Mabuza and colleagues,^23^ who refer to the trust patients have in the South African public health sector with regard to healthcare providers’ decisions. Furthermore, some patients prefer to not be involved in decision making and rather rely on the providers as the only authority.^24^

The ‘silence’ of patients can be explained from a sociological and organisational perspective in that their voices are not always duly respected and recognised by healthcare providers,^25^ leaving little room for meaningful information-seeking discussions. Longtin *et al.*^26^ contend that the nature of patient participation is a reflection of societal norms and the culture of an organisation, in our case a public hospital complex. In a context where there is a cultural expectation that patients should play a passive role, it is not surprising that they will be more reluctant to initiate active participation. The organisational and system pressures on healthcare providers evident in this study did not support opportunities for sufficient interaction, despite patients’ rights to information on diagnosis, treatment options, benefits, risk and costs and to participation in decisions pertaining to their health, as set out by the South African Patients’ Charter^27^ and the *National Health Act* 61 of 2003.^28^

Another factor affecting patient participation is potential language barriers within multilingual settings. However, in our study we did not explicitly interrogate the role and extent of the actual and/or perceived language barriers. According to Dauer *et al.*,^29^ it is important to evaluate a patient’s level of understanding during a consultation. This was rarely observed in our study. It is difficult to change established communication patterns,^26^ as consultations often focus on moving towards closure.^30^ With diagnostic imaging, communication patterns are even more complex. Where technology forms part of the interaction process, there is a tendency toward ‘objectification of a patient’s body’ (p. 172)^31^ that may limit the social interaction between patients and the radiographers and radiologists performing the procedures.

Much has been reported on patient experiences of diagnostic imaging investigations, including their anxiety regarding an uncertain future.^32,33^ Our study found that the absence of appropriate information often resulted in patient uncertainty and anxiety about the imaging investigation itself and the role of the investigation in the treatment of their condition. According to Van Ravesteijn *et al.*,^11^ the quality and amount of information given to patients before ordering the diagnostic investigation is likely to have a reassuring effect.

In the current study, patients were often kept in suspense about the imaging results. They were unsure who would communicate the results and at which point the communication should take place. Furthermore, the patients’ journeys through the health system exhibited several points of disjointed communication, even lack of communication, which may have significantly contributed to their fears and anxieties.

There should be a balance and an interdependence between healthcare-provider responsibilities and the responsibility of patients to seek information and clarification where they have not understood.^23,34^ Such interrelatedness and interdependence should also characterise the interactions amongst healthcare providers within the organisation itself. Technical interconnectedness in the diagnostic imaging context is an essential attribute of any quest to achieve the desired outcomes. Therefore, we propose that the communication and interaction processes where diagnostic imaging investigations are involved should be based on a provider–patient-centred approach mediated through (and in some instances shaped by) technology.

### Recommendations

Because of the complex nature of interactions and communication processes, more feasibility studies are needed, using interventions at various points of contact. Recommendations and interventions to improve patient communication regarding diagnostic imaging investigations are the following:

- More effort by healthcare providers to probe patients’ level of understanding of important information.^29,35^ The teach-back method is an evidence-based method often used in health education.^36,37^
- Pamphlets and visual aids, such as posters and videos, on what to expect from diagnostic imaging investigations in general (routine referrals) or from a specialised investigation.^38^
- Special efforts to ensure that patients and parents understand the implications of specialised investigations for which signed consent is required.^38^ An appropriate information leaflet or video explaining the specific investigation – benefits, risks, procedures and process – is essential. Healthcare providers may need training in adequate counselling techniques for obtaining signed consent.
- Short questionnaire to patients prior to a consultation or an imaging investigation to explore their desires and preferences for participation or to ensure that their concerns have been covered.^39^
- Availability of a radiographer to answer questions of patients waiting for an investigation to improve patients’ comprehension of procedures and relieve anxiety and fear.
- More emphasis in undergraduate training of doctors, radiographers and nurses in explaining diagnostic imaging investigations and elicit patient participation, for example, including appropriate scenarios in practical training and in examinations.

### Limitations

This study had a number of limitations. It was conducted in only one public healthcare setting, where a small number of patients were shadowed along their journey through the healthcare system. The findings are therefore not generalisable, although the supportive verbatim accounts provided in the above sections may enhance transferability to similar settings. Patient exit interviews were less informative than expected; once the patients’ healthcare concern had been solved or attended to they were less willing to commit additional interview time to the field researcher. Some of the logistical and language constraints have already been alluded to in earlier sections.

## Conclusion

Several studies have been conducted on doctor–patient consultations and interactions at specific points of contact within the health system. The unique contribution of the current study is that it followed individual patients through various points of contact in healthcare provision, from admission to ultimate discharge or ward admission. To the best of our knowledge, this was the first study with diagnostic imaging investigations as its focal point, with the casualty and outpatient departments as point of departure.

This study could serve as a basic framework to facilitate the interactional coherence between the medical consultation, a referral for a diagnostic imaging investigation, the imaging investigation itself, the decision-making process and interactive communication of information from a South African perspective. This could contribute to a better understanding of the broader medical encounter that expands beyond the dyadic doctor-patient consultation. The referral for a diagnostic imaging investigation and the encounter with providers and technology at the imaging department necessitates an appreciation of the complexity of patient participation and interaction with multiple healthcare providers. An awareness of the expectations and experiences of patients beyond the doctor-patient consultation as they journey through the health system is essential to achieving quality provider-patient-centred care.
